# Structure-property relations of co-doped bismuth layer-structured Bi_3.25_La_0.75_(Ti_1-*x*_Mo*_x_*)_3_O_12 _ceramics

**DOI:** 10.1186/1556-276X-7-42

**Published:** 2012-01-05

**Authors:** Pasinee Siriprapa, Anucha Watcharapasorn, Sukanda Jiansirisomboon

**Affiliations:** 1 Department of Physics and Materials Science, Faculty of Science, Chiang Mai University, Chiang Mai, 50200, Thailand; 2 Materials Science Research Center, Faculty of Science, Chiang Mai University, Chiang Mai, 50200, Thailand

**Keywords:** ceramics, X-ray diffraction, dielectric properties, microstructure, ferroelectricity

## Abstract

In this work, the fabrication and investigation of substituting higher-valence Mo^6+ ^for Ti^4+ ^ion on the B-site of La^3+^-doped Bi_4_Ti_3_O_12 _[BLT] structure to form Bi_3.25_La_0.75_(Ti_1-*x*_Mo*_x_*)_3_O_12 _[BLTM] (when *x *= 0, 0.01, 0.03, 0.05 0.07, 0.09, and 0.10) ceramics were carried out. X-ray diffraction patterns of BLTM ceramics indicated an orthorhombic structure with lattice distortion, especially with a higher concentration of a MoO_3 _dopant. Microstructural investigation showed that all ceramics composed mainly of plate-like grains. An increase in MoO_3 _doping content increased the length and thickness of the grain but reduced the density of the ceramics. Electrical conductivity was found to decrease, while the dielectric constant increased with Mo^6+ ^doping concentration. Ferroelectric properties were found to be improved with increasing MoO_3 _content and were optimized at *x *= 0.1.

## Background

In recent years, the family of bismuth layer-structured ferroelectrics has received much attention as the candidate for ferroelectric random access memories. An extensively studied bismuth titanate (Bi_4_Ti_3_O_12 _[BIT]) is a member of the Aurivillius family that can be represented by a general formula (Bi_2_O_3_)[A_*m*-1_(B)*_m_*O_3*m*+1_] which consists of (Bi_2_O_2_)^2+ ^sheets alternating with (Bi_2_Ti_3_O_10_)^2- ^perovskite-like layers [1]. BIT has large spontaneous polarization along the *a *axis ( approximately 50 μC/cm^2^), low processing temperature, high Curie temperature, and is a Pb-free material [2]. However, it still has high leakage current and domain pinning due to defects, such as Bi vacancies accompanied by oxygen vacancies [3,4]. To overcome these problems, A-site substitution by a replacement of volatile Bi with rare earth or other metal oxide additives is often necessary for ferroelectric property improvement. For example, ions of La [5], V [6], Nd [7], and Pr [8] have been used to substitute the Bi ion in a BIT bulk material or thin film without destroying its layered structure. Bu et al. [9] prepared La-doped BIT thin films by pulsed laser deposition and reported that these films were appropriate for non-volatile random access memory devices because of their high remanent polarization and low leakage current. In recent years, SimÕes et al. [10] reported that a doping content of *x *= 0.75 in Bi_4-*x*_La*_x_*Ti_3_O_12 _[BLT] showed an improvement of the fatigue endurance upon a repeated cyclic electric field which emphasized its possible use in FRAM applications. However, these BLT ceramics still showed a rather high leakage current. Attention has thus been paid to investigate this material in order to overcome this disadvantage. Wang et al. [11] reported that substitution of high-valence ions, such as Mo^6+^, in BLT thin films or known as Mo^6+ ^co-doped BLT thin films led to polarization fatigue improvement, high remanent polarization, and lower leakage current density.

In terms of ceramics, there has been no detailed study on a new co-doped bismuth-layered structure based on Mo^6+^-doped Bi_3.25_La_0.75_Ti_3_O_12_. In this study, therefore, effects of MoO_3 _doping concentration on the phase, microstructure, and electrical properties (i.e., conductivity, dielectric, and ferroelectric properties) of BLT ceramics produced by a conventional solid-state mixed-oxide method are reported and discussed.

## Methods

A perovskite bismuth-layered structure based on Bi_3.25_La_0.75_(Ti_1-*x*_Mo*_x_*)_3_O_12 _[BLTM] (*x *= 0, 0.01, 0.03, 0.05, 0.07, 0.09, and 0.10) powders was prepared using a solid-state mixed-oxide method. Starting binary oxide powders, i.e., Bi_2_O_3 _( > 98%, Fluka; Sigma-Aldrich Corporation, St. Louis, MO, USA), La_2_O_3 _(99.98%, Fluka), TiO_2 _( > 99%, Riedel-de Haën; Sigma-Aldrich Corporation, St. Louis, MO, USA), and MoO_3 _(99.9%, Fluka), were ball-milled and calcined at 750°C for 4 h. The slurry was transferred to a spherical flask and placed in a shell freezer. The flask was rotated in an ethanol bath for at least 1 h. The flask of frozen slurry was then immediately transferred to a vacuum dryer and dried for 24 h. After the ice was sublimated, fine dried powder was produced. The BLTM powders were then pressed under a uniaxial hydraulic pressure of 5.5 MPa with a few drops of 3 wt.% polyvinyl alcohol used as a binder. The pressed samples were sintered at temperatures in a range of 1,000 to 1,150°C for 4 h. Optimum sintering temperature for producing highest-density ceramics was determined, and the samples were selected for further characterization. Phases of selected ceramics were characterized using an X-ray diffractometer [XRD] (X-pert, PANalytical B.V., Almelo, The Netherlands) with CuKα radiation. Density was measured by Archimedes' method. The ceramics were polished and thermally etched at a temperature of 150°C below the optimum sintering temperature for 15 min dwell time prior to microstructural investigation using a scanning electron microscope [SEM] (JEOL JSM-6335F, JEOL Ltd., Akishima, Tokyo, Japan). Average grain size was determined using a mean linear intercept method from SEM micrographs. Electrical conductivity measurement was done at 1 kHz using an LCZ meter. Dielectric constant [*ε*_r_] and loss tangent [tan*δ*] were measured at room temperature with a frequency between 1 to 100 kHz using LCR Hitester 3532-50 (Hioki, Ueda, Nagano, Japan). Ferroelectric hysteresis polarization-electric field [P-E] loops were determined using a computer-controlled modified Sawyer-Tower circuit. Remanent polarization [*P*_r_], maximum polarization [*P*_max_], coercive field [*E*_c_], maximum field [*E*_max_], and loop squareness [*R*_sq_] values were evaluated from the loops.

## Results and discussion

**Figure 1:**
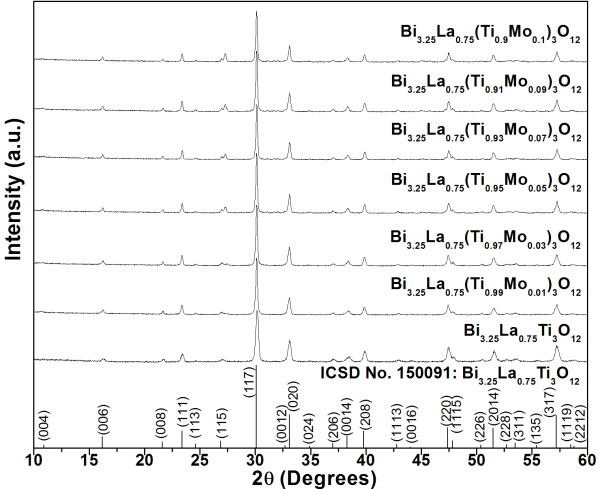
**X-ray diffraction patterns of BLTM powder**. All powders were examined in a 2*θ *range of 10° to 80° and calcined at 750°C.

**Figure 2:**
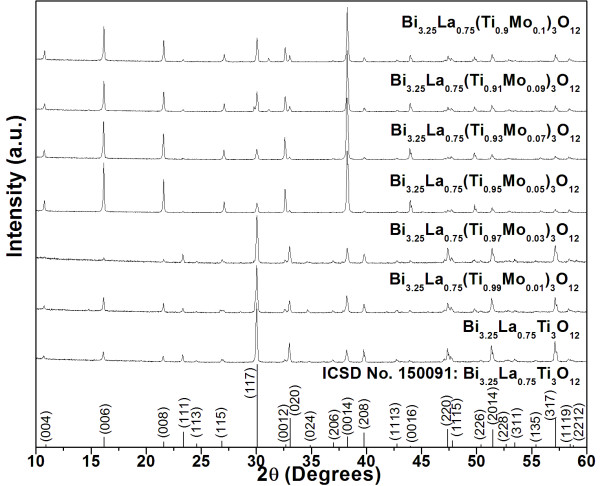
**X-ray diffraction patterns of BLTM ceramics**. All ceramics were examined in a 2*θ *range of 10° to 80° and sintered at 1,100°C.

X-ray diffraction patterns of BLTM calcined powders with various MoO_3 _doping concentration are shown in Figure 1. The patterns were well matched with the ICSD No. 150091 database of Bi_3.25_La_0.75_Ti_3_O_12_, which indicated an existence of a single orthorhombic phase without a detectable second phase. The densification data of BLTM ceramics sintered at various temperatures between 1,000 to 1,150°C (not shown here) indicated that the optimum sintering condition for achieving maximum density was 1,100°C and 4 h dwell time. These ceramic samples were selected for phase characterization whose results are shown in Figure 2. All XRD patterns basically indicated an orthorhombic structure similar to those found in the powders. It could be seen from the XRD patterns of samples with high MoO_3 _doping content (≥0.05 mol) that X-ray intensities of a particular set of planes, i.e., (004), (006), (008), (0012), (0014), and (0016), were relatively higher than those in the samples with lower MoO_3 _content. This suggested that the preferred orientation of BLT crystallites was increased due to Mo^6+ ^ions.

**Figure 3:**
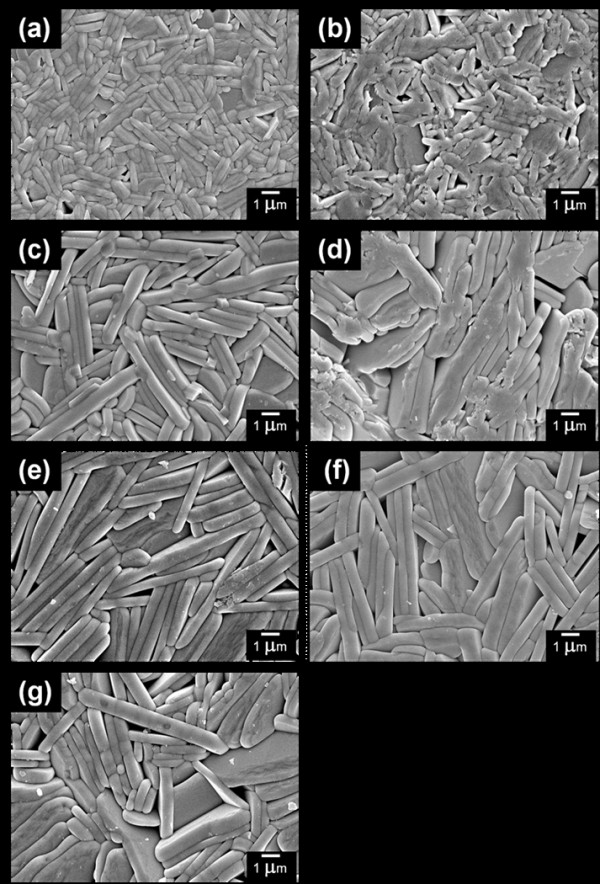
**SEM micrographs of Bi_3.25_La_0.75_(Ti_1-*x*_Mo*_x_*)_3_O_12 _ceramics**. (**a**-**g**) indicated *x *= 0, 0.01, 0.03, 0.05, 0.07, 0.09, and 0.10, respectively.

Density and grain size measured in terms of grain length are presented in Table 1. Increasing MoO_3 _doping content was found to reduce density values. Typical morphologies of polished and thermally etched surfaces of BLTM ceramics are shown in Figure 3. All samples showed plate-like morphology with variation in grain size and orientation. It was obvious that both grain length and thickness gradually increased with increasing MoO_3 _content. It was also recognized that MoO_3 _played an important role in increasing anisotropies of the surface and grain boundary energies and mobilities [12,13]. The sample containing MoO_3 _≥ 0.05 mol showed grains which tended to stack and align in the same direction. For those containing a smaller amount of MoO_3 _(≤0.03 mol), their grains became smaller with a more randomized grain orientation. This observation corresponded well to the XRD result (Figure 1b) where the preferred orientation of (00l)-type planes occurred in BLTM ceramics with high MoO_3 _concentration.

**Table 1:** Physical, dielectric, and ferroelectric properties of Bi_3.25_La_0.75_(Ti_1-_*_x_*Mo*_x_*)_3_O_12 _ceramics

Mo content (mol)	Density (g•cm^-3^)	Grain length (μm)	Dielectric properties^a^		Ferroelectric properties		
			*ε*_r_	tan*δ*	*P*_r_*/P*_max_	*E*_c_*/E*_max_	*R*_sq_
0	7.46 ± 0.03	2.59 ± 0.61	127	0.001	0.05	0.05	0.06
0.01	7.21 ± 0.01	2.66 ± 0.79	124	0.007	0.23	0.23	0.25
0.03	7.16 ± 0.03	4.61 ± 2.13	414	0.551	0.96	0.69	1.15
0.05	6.74 ± 0.02	5.94 ± 2.11	402	0.519	0.74	0.58	0.84
0.07	6.94 ± 0.02	7.73 ± 3.13	387	0.510	0.78	0.70	1.04
0.09	6.79 ± 0.01	8.04 ± 3.31	310	0.461	0.81	0.71	1.06
0.10	6.83 ± 0.02	8.13 ± 2.97	308	0.349	0.96	0.57	1.15

^a^Measurement was carried out at room temperature and at a frequency 1 kHz. *ε*_r_, dielectric constant; tan*δ*, loss tangent; *P*_r_, remanent polarization; *P*_max_, maximum polarization; *E*_c_, coercive field; *E*_max_, maximum field; *R*_sq_, loop squareness.

**Figure 4:**
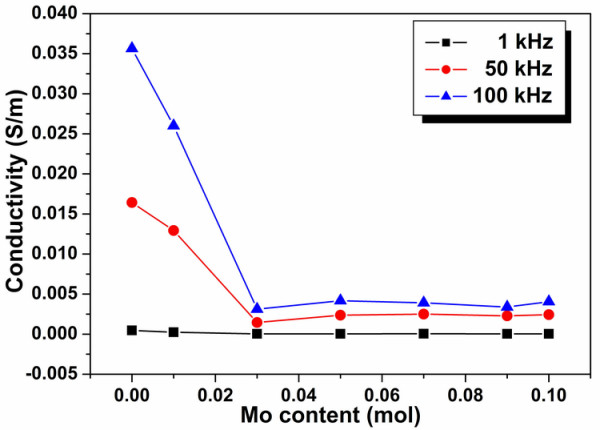
**Relationship between electrical conductivity and MoO_3 _content of Bi_3.25_La_0.75_(Ti_1-*x*_Mo*_x_*)_3_O_12 _ceramics**. The measurement was done at frequencies of 1, 50, and 100 kHz.

The electrical conductivity of BLT and BLTM ceramics measured at frequencies of 1, 50, and 100 kHz is shown in Figure 4. The conductivity values of BLTM were lower than those of undoped BLT ceramic regardless of measurement frequency. This seemed to be the effect of the donor dopant on the electrical conductivity of a p-type material in which electrons were introduced when Mo^6+ ^substituted at Ti^4+ ^site compensating holes present in the sample [14]. According to previous studies on vanadium-doped BIT [6] and niobium-doped BIT [14], oxygen vacancies [$V_{o}^{..}$] could similarly be reduced by Mo^+6 ^substitution for Ti^+4 ^site in BLT ceramics. For BLT, electrical conductivity at low frequencies, i.e., 1 and 50 kHz, was much lower than that at 100 kHz. This could be explained by the contributions of space-charge polarization at the grain boundary and ionic polarization caused by ionic motion [15-17]. It has been reported that a low-frequency conductivity response was dominated by the grain boundary, while a high-frequency conductivity response was dominated by ferroelectric crystalline layers inside the grains. Hence, it could be speculated that the low-frequency dependence was dominated by an interacting charge carrier system, which was similar to an ionic conductor. For BLTM ceramics, the apparent decrease in frequency dependence of alternating current [AC] conductivity seemed to correspond well to an increase in grain size which in turn reduced the amount of grain boundaries. Among BLTM samples whose average grain size was about the same, these ceramics therefore showed similar frequency dependence and magnitude of AC conductivity.

**Figure 5:**
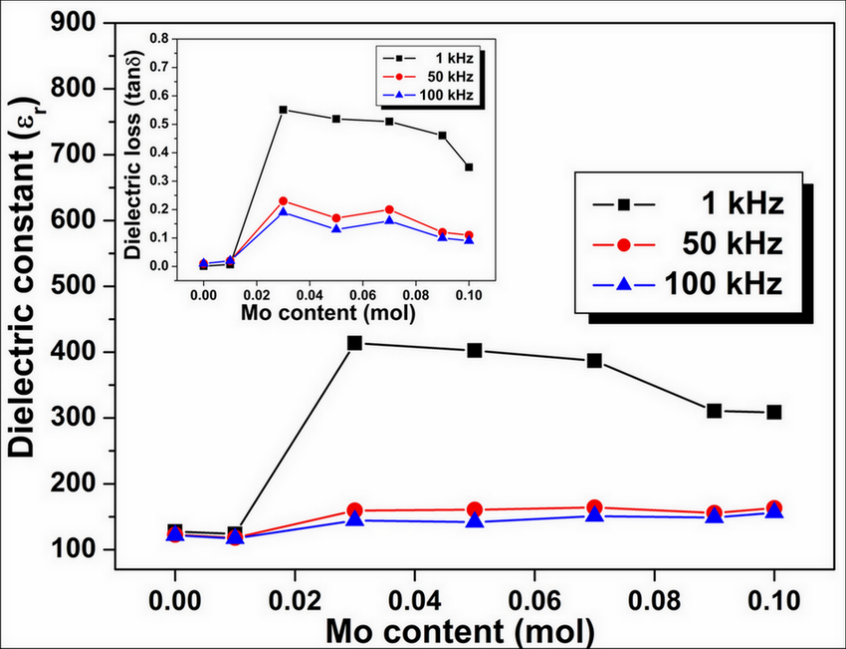
**Relationship between dielectric constant and MoO_3 _content of Bi_3.25_La_0.75_(Ti_1-*x*_Mo*_x_*)_3_O_12 _ceramics**. The measurement was done at frequencies of 1, 50, and 100 kHz. The inset shows dielectric loss tangent as a function of MoO_3 _content.

Room temperature *ε*_r _and dielectric tan*δ *of the BLTM ceramics are shown in Figure 5, and the values are listed in Table 1. It was experimentally observed that the dielectric constant gradually increased from 127 for BLT to a maximum of 414 for 0.03 mol MoO_3 _doping content. An increase in dielectric constant of BLTM was expected to be caused by partial substitution of Mo^6+ ^ion for Ti^4+ ^ions in the B-site which implied that the MoO_3 _dopant reduced space charges or ionic conduction and resulted in a low leakage current which, in turn, resulted in an enhanced dielectric constant. A further increase in MoO_3 _content over 0.03 mol was found to decrease the dielectric constant, possibly due to overcompensation of charge which caused a slight increase in electrical conductivity.

**Figure 6:**
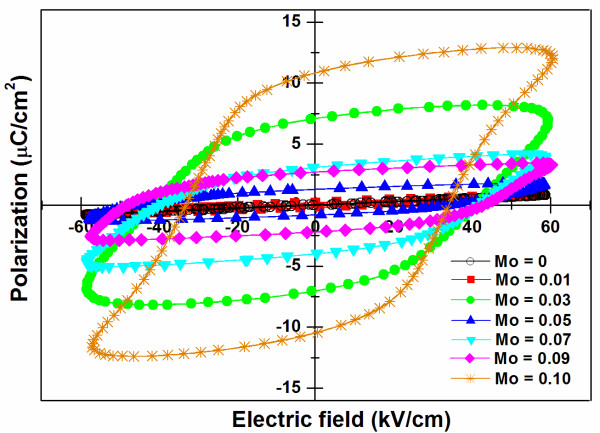
**Relationship between P-E hysteresis loops and MoO_3 _content of Bi_3.25_La_0.75_(Ti_1-*x*_Mo*_x_*)_3_O_12 _ceramics**. The measurement was done at a frequency of 50 Hz for BLTM ceramics sintered at 1,100°C.

Ferroelectric P-E hysteresis loops of MoO_3_-doped BLT ceramics were measured at room temperature with an applied field of 60 kV/cm and a frequency of 50 Hz as shown in Figure 6. For undoped BLT, no hysteresis behavior was observed. It was previously reported by Zhang et al. [17] that an electric field as high as 530 kV/cm was required to pole a BLT thin film at room temperature. In addition, Kim et al. [18] showed that hysteresis behavior was observed when the polarization was carried out using an applied field of approximately 60 kV/cm at 50°C. Therefore, it seemed that a relatively high leakage current as seen from its high electrical conduction was the main reason for the non-hysteresis behavior observed in the BLT ceramic. Remanent polarization and coercive field were found to increase after Mo^6+ ^was doped into BLT ceramics which were similar to a previous investigation on Nb-doped BLT ceramics [18]. Because of the temperature and field dependence of ferroelectric properties, i.e., remanent polarization and coercive field of ceramics [19,20], these parameters were normalized in a form of *P*_r_*/P*_max _and *E*_c_*/E*_max _values, where *P*_max _was the polarization value at the *E*_max_. These ratios are listed in Table 1 along with the values of *R*_sq_. It seemed that Mo^6+ ^substituting for Ti^4+ ^in the BLT ceramic efficiently decreased the concentration of $V_{o}^{..}$, which weakened the influence of domain pinning on polarization [21]. A decrease of space charge ($V_{o}^{..}$) density as well as electrical conductivity thus led to the large remanent polarization observed. In addition, changes in domain structures may be one of the factors affecting the coercive field [14]. As can be seen from the figure, the BLTM ceramic with a Mo doping concentration of 0.1 mol showed a well defined P-E hysteresis loop and better squareness than BLT and other BLTM ceramics. This research thus suggested that the partial substitution of Mo^6+ ^for Ti^4+ ^ions at the B-site could improve ferroelectric properties of BLT ceramics with an optimum ferroelectric behavior obtained at *x *= 0.1 mol.

## Conclusions

A new system of co-doped bismuth titanate-layered structure ceramics, i.e., Bi_3.25_La_0.75_(Ti_1-*x*_Mo*_x_*)_3_O_12 _or BLTM (*x *= 0, 0.01, 0.03, 0.05, 0.07, 0.09, and 0.10), was successfully prepared by a solid-state mixed-oxide method. X-ray diffraction analysis indicated that the MoO_3 _dopant induced a preferred orientation of the BLT ceramics with changes in lattice constant of its orthorhombic structure. Grain size was found to increase with increasing MoO_3 _doping content. Electrical conductivity of BLTM was slightly decreased by Mo^6+ ^donor doping due to the charge compensation between the provided excess electrons and inherent holes. This led, consequently, to an increased dielectric constant from 127 for BLT to a maximum of 414 for 0.03 mol MoO_3 _doping content. Addition of MoO_3 _content also increased the remanent polarization of over 10 μC/cm^2^, and this value was shown to be much larger than that of the BLT ceramic, due to a reduction of domain pinning oxygen vacancies and leakage current loss with optimum ferroelectric properties obtained for the composition of *x *= 0.1 mol. It is suggested that molybdenum-doped BLT could be an alternative material for potential applications in electronic industries which require a lead-free material having large remanent polarization and lower processing temperatures.

## Competing interests

The authors declare that they have no competing interests.

## Authors' contributions

PS carried out the BLTM ferroelectric ceramic experiments, analysis, and writing of the manuscript. AW and SJ participated in the conception and design of the study and revised the manuscript for important intellectual content. All authors read and approved the final version of the manuscript.
